# The association between types of seafood intake and the risk of type 2 diabetes: a systematic review and meta-analysis of prospective cohort studies

**DOI:** 10.15171/hpp.2019.24

**Published:** 2019-08-06

**Authors:** Nazli Namazi, Neil R. Brett, Nick Bellissimo, Bagher Larijani, Javad Heshmati, Leila Azadbakht

**Affiliations:** ^1^Diabetes Research Center, Endocrinology and Metabolism Clinical Sciences Institute, Tehran University of Medical Sciences, Tehran, Iran; ^2^School of Nutrition, Ryerson University, Toronto, Ontario, Canada; ^3^Endocrinology and Metabolism Research Center, Endocrinology and Metabolism Clinical Sciences Institute, Tehran University of Medical Sciences, Tehran, Iran; ^4^Department of Nutritional Science, School of Nutritional Science and Food Technology, Kermanshah University of Medical Sciences, Kermanshah, Iran; ^5^Department of Community Nutrition, School of Nutritional Sciences and Dietetics, Tehran University of Medical Sciences, Tehran, Iran; ^6^Department of Community Nutrition, School of Nutrition and Food Science, Isfahan University of Medical Sciences, Isfahan, Iran

**Keywords:** Seafood, Diabetes mellitus, Type 2, Fatty acids, Meta-analysis

## Abstract

**Background: ** Seafood is the main source of long-chain n-3 polyunsaturated fatty acids (n-3PUFAs) with beneficial health effects; however, findings on the association between the consumption of different types of seafood and type 2 diabetes mellitus (T2DM) are conflicting. Our objective was to perform a systematic review and meta-analysis examining the relationship between different types of fish/seafood and the risk of T2DM in adult populations.

**Methods:** A systematic search of PubMed/Medline, Scopus, and Web of Science (ISI) databases was performed for cohort studies, published in English, before 1 September 2017. Multivariate adjusted relative risk (RR) estimates with 95% confidence intervals (CIs) for each category of seafood were pooled to examine the association.

**Results:** Comparing the highest vs. lowest fatty fish intake categories indicated that there was a significant inverse association between the consumption of fatty fish and onset of T2DM (RR:0.89; 95 % CI: 0.82, 0.98; I^2^: 0%, P=0.54). However, after performing sensitivity analysis, we found that eliminating one study resulted in a non-significant association (RR: 0.93; 95 % CI:0.80, 1.09). There were no significant associations between lean fish (RR: 1.03; 95% CI: 0.87,1.22, I2: 51.0%, P=0.08), seafood other than fish (RR: 0.95; 95% CI: 0.83, 1.10, I^2^: 71.2%,P=0.002), fish products (RR: 0.96; 95% CI: 0.82, 1.13, I^2^:0%, P=0.62), and fried fish (RR: 1.02;95% CI: 0.83, 1.26, I^2^:71.2%, P=0.06) and T2DM risk.

**Conclusion:** The risk of T2DM was not associated with the intake of lean fish, seafood other than fish, and fish products. However, due to the low robustness of findings regarding protective roles of oily fish, more longitudinal studies are needed to clarify this association.

## Introduction

Type 2 diabetes mellitus (T2DM) accounts for at least 90% of all diabetes cases and the global prevalence of T2DM has reached alarming levels, having more than doubled over the past 30 years.^1^ T2DM increases the risk of many serious diseases or conditions, including coronary heart disease, kidney failure and retinopathy. Further, the rates of T2DM have been rapidly rising in children and adolescents.^2^ Thus, prevention of T2DM is a top public health concern, with major strategies for prevention targeting weight management and dietary modification.^3,4^ According to a consensus statement from the International Diabetes Federation, dietary factors important for T2DM prevention include foods that lower glycemic response and having regular intake of n-3 polyunsaturated fatty acids (n-3 PUFAs).^4^ Fish and other seafood are complete protein sources that can lower glycemic response of a meal and are also the predominant dietary source of n-3 PUFAs.^5^

Though the amounts of n-3 PUFAs varies greatly among types of fish and seafood, previous cohort studies of total fish or seafood intakes in adults have observed inverse associations between fish/seafood and the risk for the development of T2DM.^6-9^ Dietary intake of n-3 PUFAs has been shown to decrease systemic inflammatory markers, circulating blood lipids and lowering risk of T2DM.^4^ Interestingly however, four cohort studies examining intakes of n-3 PUFA rich seafood reported positive associations between n-3 PUFAs and the development of T2DM.^5,10-12^ It is possible that environmental contaminants present in fish are causing this reported relationship of n-3 PUFA intake and T2DM.^10^ Recent work from Canada demonstrated that environmental contaminants present in fish (dichlorodiphenyldichloroethylene [DDE] and polychlorinated biphenyls [PCBs]) positively associated with risk of T2DM (odds ratio [OR] = 1.09 [95% CI: 1.05-1.75] for DDE and OR = 1.07 [95% CI: 1.004-1.27] for PCBs), whilst n-3 PUFA intake adjusted for DDE/PCBs had an inverse association with T2DM (OR = 0.86 [95% CI: 0.46-0.99]).^10^ Since concentrations of environmental pollutants are biomagnified when moving up the food chain, considering types of seafood consumed is vital when examining the relationship between fish/seafood intake and T2DM.^1,11^

There are several systematic reviews on the association between fish consumption and the risk of diabetes; however, only two systematic reviews examined the association of different types of fish and diabetes that they had some limitations. Though Muley et al revealed that higher intake of oily fish reduced the incidence of T2DM, importantly, the effects of other types of fish and marine animals were not considered.^13^ In a meta-analysis by Zhang et al, in 2012, it was reported that the consumption of oily fish can lower the risk of T2DM, while no significant association was found for lean fish.^14^ However, this meta-analysis had significant study heterogeneity and did not consider methodological quality. Additionally, they did not examine the association of fish products, seafood other than fish and fried fish and T2DM. The aforementioned meta-analyses also did not evaluate the robustness of findings and publication bias. Accordingly, our objective was to perform a systematic review and meta-analysis examining the relationship between different types of fish/seafood and the risk of T2DM in adult populations.

## Materials and Methods

Adhering to the guidelines of the Preferred Reporting Items for Systematic Reviews and Meta-Analysis Statement (PRISMA),^15^ a systematic literature search for articles published in English up to 31 August 2017 was performed using PubMed/Medline, Scopus and Web of Science (ISI). Additionally, to avoid missing relevant publications, reference lists of retrieved papers were assessed by hand.

### Search strategy

Keywords that were used for the primary search strategy were both medical subheadings (MESH) and free terms. Search terms contained ‘fish’, ‘seafood’, ‘diabetes’ ‘diabetic’, ‘T2DM’, and ‘NIDDM’. PICOS criteria are presented in Supplementary file 1.

### Eligibility criteria

The inclusion criteria were as follows: (*i*) prospective cohort study; (*ii*) the exposure of interest was different types of fish or any other seafood products; (*iii*) the outcome was T2DM, (*iv*) the study population did not suffer from diabetes at baseline, and (*v*) reporting multivariate adjusted relative risk (RR) estimates with 95% CI for each category of fish/seafood. Publications were excluded if: (*i*) they were cross-sectional studies, clinical trials, case-control, case reports, case series, or *In vitro*/ animal models; (*ii*) examined total fish consumption or n-3 fatty acids supplement; (*iii*) include patients with diabetes at baseline, children or athletes; and (*iv*) examined other types of diabetes besides T2DM. Conference abstracts, theses, books, and publications with non-English languages were excluded as well. Each identified publication was independently examined by two reviewers (N.N, J.H) to determine whether it was eligible for inclusion. Disagreements between the two reviewers were resolved by discussion to reach consensus or by principal investigator (B.L).

### Data extraction

The following characteristics were extracted from the eligible papers by two reviewers (N.N, J.H) independently: the first author’s name, year of publication, country, sex, mean age at baseline, sample size, duration of follow-up and person-years, number of cases, methods for dietary assessment, seafood category (oily fish, lean fish, fish products, other seafood, fried fish, etc.), seafood intake, frequency of seafood consumption, variables that were adjusted in the analysis as well as RRs and 95% CIs for the highest vs. lowest categories of each type of seafood.

When studies reported findings from different covariate analyses, only the model that contained the most potential confounders was extracted. As the purpose of the present meta-analysis was to examine the link of different types of seafood, not seafood in total, if results were reported for both total fish/seafood and the type of seafood, only the results for each type of seafood were extracted. When more than one study extracted from the same cohort study was published, we included the newer study. Any disagreements were resolved by a third reviewer (L.A).

### Risk of bias assessment (quality assessment)

Risk of bias was assessed by two independent reviewers (N.R.B, N.B) using the Newcastle-Ottawa Scale adapted for cohort studies.^16^ The Newcastle-Ottawa Scale included three sections as follows: the selection of study groups (0-4 stars), adequacy of adjustment for confounding (0-2 stars), and ascertainment of the outcome of interest (0-3 stars). Thus, the maximum score for this scale is 9. If any paper received a score of ≥7 stars, it was considered to have a low risk of bias (high quality study), otherwise it was categorized with a high risk of bias (low quality study). Any controversies were resolved by the principal investigator (B.L).

### Data synthesis and statistical analysis

The extracted effect sizes in the current meta-analysis were RRs and 95% CIs for the risk of T2DM onset in people who had the highest consumption of fish/other seafood compare to those with the lowest intakes. The effect sizes were pooled by the method of DerSimonian and Laird using random effects.^17^ Between-study heterogeneity was examined using I^2^statistics. I^2^ values >50% was considered high heterogeneity.^18^ Subgroup analyses were performed for each type of fish/seafood to identify either the main sources of heterogeneity or examine the effects of each parameter on the results. Wherever possible (existence of a minimum of two studies in each category), stratification was done using the following parameters: country (European vs. Asian), gender (men, women, both), dietary assessment tool (food frequency questionnaire [FFQ], 24-hour recall/ other questionnaires), Body mass index (BMI) categories (overweight, normal weight), and study quality (less, equal or more than 7). Sensitivity analysis was used to elucidate the robustness of the pooled effect size after the removal of an individual study from the analysis. Egger’s regression asymmetry test was used to examine publication bias as <10 studies were included in the meta-analysis. All statistical analyses were carried out using Stata, version 11.0 (Stata Corp, College Station, TX). *P* values <0.05 were considered statistically significant.

## Results

### Literature search

**Figure 1:**
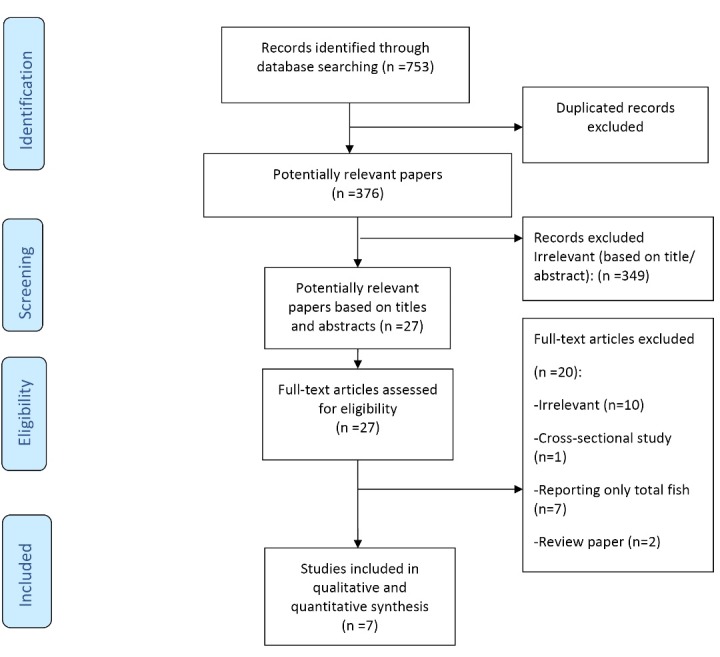
‏ PRISMA flowchart for study identification and selection‏.

In total, 753 publications were identified in PubMed (n = 246), Scopus (n = 216) and Web of Knowledge (n = 291) searches, of which 377 were duplicates. As presented in Figure 1, after the removal of duplicates and an initial screen of titles and abstracts, 27 publications were potentially relevant. After careful examination, 20 studies were further excluded due to: not being relevant (n = 10), reporting only total fish instead of different types of fish/seafood (n = 7), cross-sectional study (n = 1), and review papers (n = 2).

### Study characteristics

Overall, 7 cohort studies^6-9,19-21^ were identified and included in the current meta-analysis. Characteristics of the included studies were summarized in Table 1. Studies were published between 2009 and 2017. They were conducted in European (n = 5)^19-21^,and Asian (n = 2)^6,9^ countries. One prospective study^7^ reported the effect sizes for 8 European countries including France, Italy, Spain, UK, the Netherlands, Sweden, Denmark, Germany, separately. Sample sizes varied between 4472 and 51 963. Five of the six studies included adults populations >50 years of age (range: 51-67 years), with the remaining study^8^ having mean ages <50 years. The included studies either examined the association of seafood intake and the incidence of T2DM in both genders, separately (n = 2),^6,9^ in combination (n = 3),^7,19,21^ or only in men^20^or only in women.^8^ Data on dietary assessment were collected using FFQs (n = 5),^6,8,9,20,21^ quantitative dietary questionnaires (n = 1)^7^or 24-hour dietary recalls (n = 1).^19^ The mean BMI for studies was 23.0-26.5 kg/m^2^. The score of all prospective cohort studies were more than the mean score of Ottawa checklist (range: 6 to 8 stars) and four included studies had high quality (score ≥7). All studies except one^8^ provided adjusted risk estimates for total energy intake. Additionally, among 7 included studies, only one^19^ was not adjusted for BMI. Person-years were not reported for all studies. Based on available data, it varied between 46 7961^20^and 3 990 000.^7^

**Table 1:** Main characteristics of the included studies on the association between different types of fish and the incidence of type 2 diabetes

**First Author (year)**	**Country**	**Age range**	**Gender**	**Sample size**	**Cases**	**Duration follow-up (y)**	**Exposure/Assessment tool**	**Outcome (ascertainment)**	**Comparison**	**Adjustments** ^*^	**Quality score**
Wallin et al (2017)	Sweden	60	M	35583	3624	15	FFQ	Linkage of the study cohort with the Swedish National Diabetes Register (NDR) and the Swedish National Patient Register (NPR)	≥6 vs.<1 serving/month	1, 2, 3, 4, 5, 6, 7, 8, 9	8
							Fried fish				
							Seafood other than fish		≥1 serving/week vs. never		
Rylander et al (2014)	Norway	47	F	33261	479	NR	FFQ	Self-reported	50 vs.0 g/day	1, 2, 3, 5, 10	6
							Oily fish				
							Lean fish		100 vs.0 g/day		
							Fish products		100 vs.0 g/day		
Patel et al (2012)	France	51	Both	2684	12403		Quantitative dietary assessment and 24-h dietary recall	Multiple data sources including: self-report of doctor-diagnosed diabetes, record linkage including listing with general practice diabetes registers, regional hospital outpatient diabetes registers, and hospital admissions information	>102.6 vs.≤ 4.1 g/week	2, 3, 4, 5, 6, 7, 11, 12	7
	Italy										
	Spain										
	UK										
	Netherlands			2661			Lean fish		>139.7 vs.0		
	Sweden										
	Denmark			2253			Seafood other than fish		>30.3 vs.0 g/week		
	Germany										
Nanri et al (2011)	Japan	56	M	22921	572	NR	FFQ	Self-reported	Q4 vs.Q1	1, 2, 3, 5, 6, 7,10, 13, 14	6
							Oily fish				
							Lean fish				
							Seafood other than fish				
							Fish products				
Villegas et al (2011)	China	52	M	51963	NR	NR	FFQ	Self-reported; American Diabetes Association criteria	Q5 vs. Q1	1, 2, 3, 4, 5, 6, 7, 10, 13, 16, 17	6
		49	F				Seafood other than fish				
Woudenberg et al, (2009)	Netherlands	67	Both	4472	463	15	24-h dietary recall	American Diabetes Association criteria and the World Health Organization	≥7 g/d vs. no intake	1, 4, 5, 6, 7, 11, 19, 20	8
							Oily fish				
							Lean fish		23 g/d vs. no intake		
Patel et al (2009)	Norwich England	58	Both	21984	725	10.2	Fried fish	Self report of doctor-diagnosed diabetes Registries	≥ vs.< 1 portions/wk	1, 2, 3, 4, 5, 6, 7, 11, 13, 22, 23	7
							Fish fingers				
							Fish roe				

NR: not reported; *1= age, 2=body mass index, 3= physical activity, 4=education, 5= smoking, 6=total energy intake, 7= alcohol intake, 8= DASH components, 9= environmental contaminants, 10=hypertension, 11= sex, 12= fruit and vegetable intake, 13= family history of diabetes, 14=coffee intake, 15=other food intake, 16=income, 17=job, 18= dietary pattern, 19=trans fatty acid, 20= dietary fiber, 21=selenium, vitamin D and cholesterol intake, 22=waist circumference, 23= plasma vitamin C

### Findings of meta-analysis

*The association of oily fish consumption and risk of T2DM*

**Figure 2:**
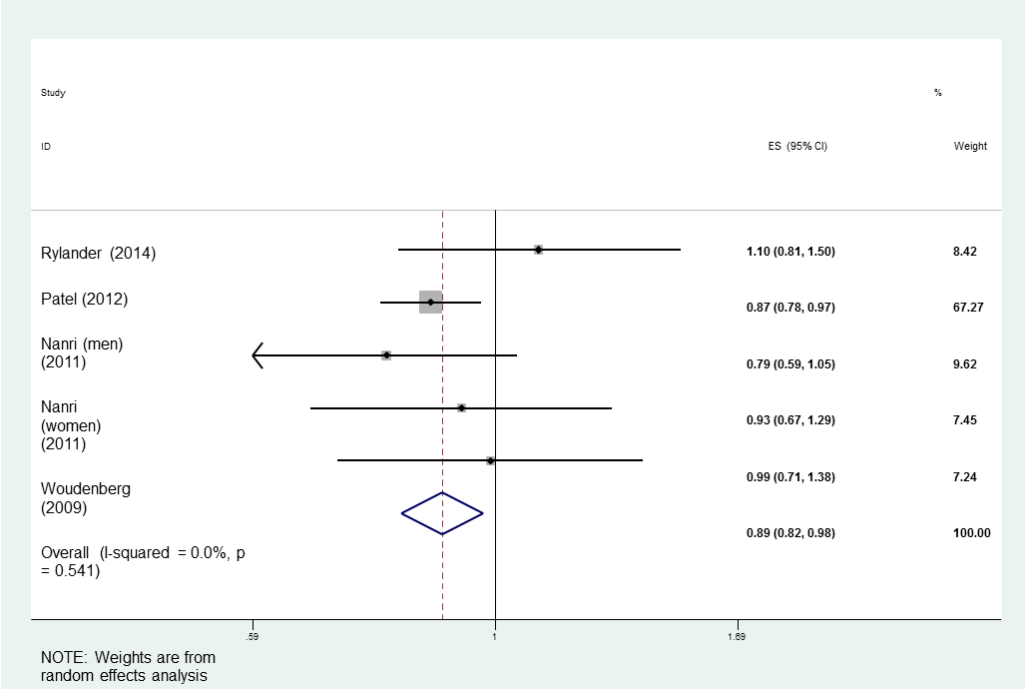
The association between oily fish consumption and the incidence of type 2 diabetes in adults.

Four studies^6-8,19^ were included to clarify the association of oily fish intake with T2DM risk. As presented in Figure 2, there was a significant inverse association with the consumption of fatty fish and T2DM (RR: 0.89; 95% CI 0.82, 0.98; I^2^: 0%; *P* = 0.54). Stratification by study quality indicated an inverse significant association between oily fish and the risk of T2DM (RR: 0.88; 95% CI: 0.79, 0.98; I^2^: 0%; *P* = 0.46), while it was not significant in low quality ones (RR: 0.93; 95% CI 0.76, 1.12; I^2^: 15.5%; *P* = 0.30) (Table 2).

*The association of lean fish consumption and risk of T2DM*

**Figure 3:**
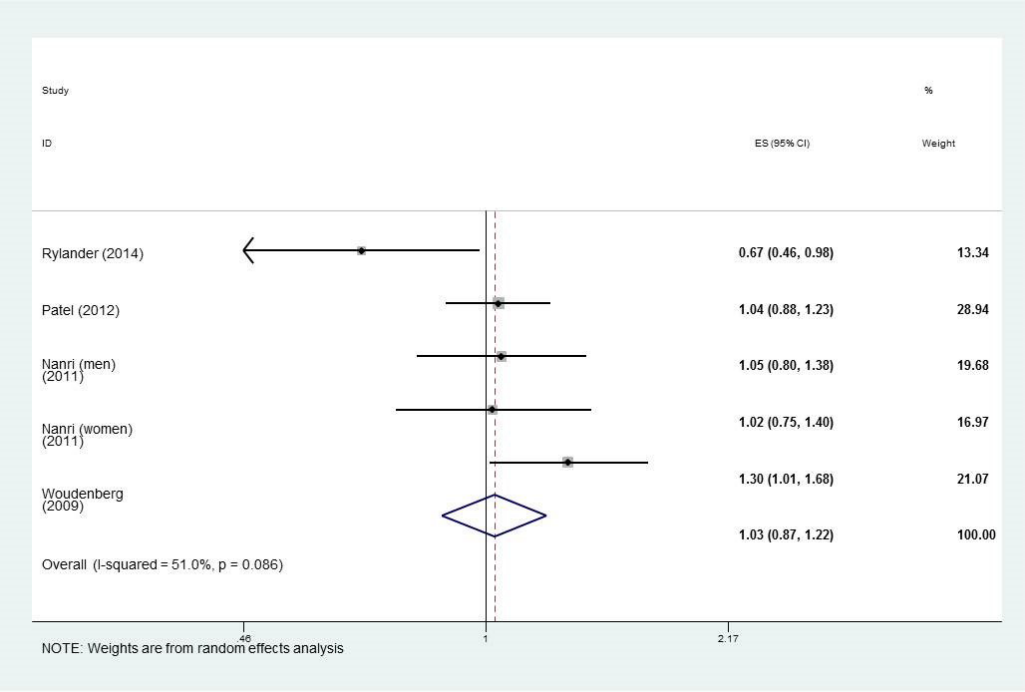
The association between lean fish consumption and the incidence of type 2 diabetes in adults.

Findings from 4 studies^6-8,19^ indicated no significant association between lean fish intake and the risk of T2DM (RR: 1.03; 95% CI: 0.87, 1.22, I^2^:51.0%, *P* = 0.08) (Figure 3). After excluding one study in Asian populations,^6^ the pooled RR did not change (RR: 0.99; 95% CI: 0.74, 1.35, I^2^:75.4%, *P* = 0.01) for European countries. Subgroup analysis based on study quality (high quality studies) showed that lean fish increased the risk of T2DM by 14%, while it was not significant (Table 2).

*The association of seafood other than fish consumption and risk of T2DM*

**Figure 4:**
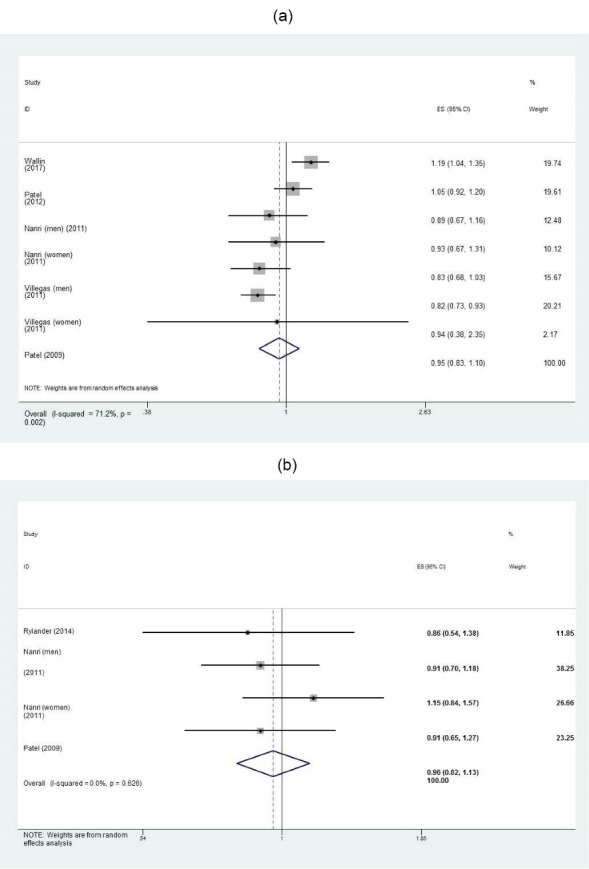
The association between intakes of a) seafood other than fish, and b) fish products and the incidence of type 2 diabetes in adults.

A meta-analysis of 5 studies^6,7,9,20,21^ revealed that the risk of T2DM in subjects with the greatest consumption of seafood other than fish was not significantly different than those with the lowest intake (RR: 0.95; 95% CI: 0.83, 1.10, I^2^: 71.2%, *P* = 0.002) (Figure 4a). To explore the source of between-study heterogeneity, we performed subgroup analysis based on a number of covariates. Stratification by countries, study qualities and BMI at baseline resulted in the greatest attenuation of heterogeneity. Subgroup analysis by country showed that seafood other than fish was protective against T2DM onset in Asian populations (RR: 0.84; 95% CI: 0.76, 0.92, I^2^: 0%, *P* = 0.87); but a positive association was observed in European populations (RR: 1.12; 95% CI: 1.02, 1.23, I^2^: 0%, *P* = 0.39). Additionally, being normal weight was protective against T2DM (RR: 0.84; 95% CI: 0.76, 0.92, I^2^: 0%, *P* = 0.87) (Table 2). Stratification by sex revealed that there was an inverse association between seafood other than fish and T2DM risk in women (RR: 0.83; 95% CI: 0.74, 0.93, I^2^: 0%, *P* = 0.48). However, the link was not significant among men (Table 2).

**Table 2:** Subgroup analysis for the association of types of seafood and the risk for type 2 diabetes

**Exposure**	**No. effect size**	**Pooled effect size (95% CI)**	**I** ^ 2 ^ **(%)**	***P***_heterogeneity_
**Oily fish**				
**Study quality**				
Equal or more than 7	2	0.88 (0.79, 0.98)	0	0.46
Less than 7	3	0.93 (0.76, 1.12)	15.5	0.30
**Lean fish**				
**Study quality**				
Equal or more than 7	2	1.14 (0.92, 1.41)	51.5	0.15
Less than 7	3	0.92 (0.71, 1.19)	49.7	0.13
**Seafood other than fish**				
***Sex***				
Men	3	0.97 (0.75, 1.25)	79.7	0.007
Women	2	0.83 (0.74, 0.93)	0	0.48
**Country**				
European	3	1.12 (1.02, 1.23)	0	0.39
Asian	4	0.84 (0.76, 0.92)	0	0.87
**Study quality**				
Equal or more than 7	3	1.12 (1.02, 1.23)	0	0.39
Less than 7	4	0.84 (0.76, 0.92)	0	0.87
**BMI**				
Overweight	3	1.12 (1.02, 1.23)	0	0.39
Normal weight	4	0.84 (0.76, 0.92)	0	0.87

*The association of fish product consumption and risk of T2DM*

The pooled effect size showed no link between the consumption of fish products^6,8,21^ and T2DM risk (RR: 0.96; 95% CI: 0.82, 1.13, I^2^: 0%, *P* = 0.62) (Figure 4b).

*The association of fried fish consumption and risk of T2DM*

Fried fish were examined in only two studies.^20,21^ In subjects who consumed the highest amount of fried fish, the risk for T2DM did not increase compare to those with the lowest intake (RR: 1.02; 95% CI: 0.83, 1.26, I^2^: 71.2%, *P* = 0.06). Though there was heterogeneity, with only two studies, no subgroup analysis was possible.

*Sensitivity analysis*

Sensitivity analysis revealed the robustness of findings in all the study variables except oily fish. After excluding Patel et al study^7^ which covered 67% of weight of the pooled studies, we observed no significant association between oily fish intake and the risk of T2DM.

*Publication bias*

There was no publication bias for the association of oily fish (*P* = 0.42), lean fish (*P* = 0.36), other seafood other than fish (*P* = 0.46), and fish products (*P* = 0.81) with the risk for T2DM (using Egger test).

## Discussion

The current meta-analysis indicated that oily fish may have protective effects against the development of T2DM. However, the robustness of findings was influenced by one of the included cohort studies. Therefore, results should be interpreted with caution. Further, other seafoods can reduce the risk of developing T2DM in Asian populations, women and normal weight individuals by 16%-17%. The consumption of lean fish, fried fish and fish products did not significantly affect the risk of T2DM. It is possible that these findings in lean fish, fried fish, and fish products are due to smaller numbers of studies or moderate heterogeneity among studies. Our findings are helpful for nutritionists and other health providers for nutritional recommendations.

Our meta-analysis suggests that oily fish intake may have a protective affect against the risk of developing T2DM. These findings were in line with previous meta-analyses that found the highest consumption of oily fish vs. the lowest one reduced the risk of T2DM.^13,14^ However, the findings were influenced by Patel et al study.^7^ As a considerable weight of the pooled effect estimates was dedicated to the aforesaid study, it was determinative in the overall finding. The weakness points of the prior meta-analyses in this regard was that sensitivity analysis was not performed and they concluded that oily fish had protective roles against T2DM. Due to the lack of robustness, we could not draw a certain conclusion about the association of oily fish and T2DM and further studies are needed.

Oily fish, including salmon, herring and mackerel are some of the richest sources of long chain n-3 PUFAs EPA and DHA. Long chain n-3 PUFAs may have a protective role against T2DM due to anti-inflammatory properties acting to decrease pro-inflammatory cytokine and NF-κB production,^22^ and stimulate PPAR-gamma receptors.^23^ However, a meta-analysis looking at long chain n-3 PUFA supplementation, found it had no benefit on the risk of T2DM.^24^ This suggests that n-3 PUFAs consumed with other nutrients in fish may lower risk of T2DM. First, proportions of amino acids may differ among types of fish, for example, the concentration of taurine, in cod is greater than farmed salmon. Evidence indicated an inverse association between taurine, diabetes^25^ and CVD.^26^ Oily fish are also a rich source of vitamin D, and a number of recent cohort studies have suggested that vitamin D status in both children and adults in inversely associated with risk of T2DM.^27,28^ Every 15 nmol/L increase in vitamin D status decreased the odds of T2DM, impaired fasting glucose and high hemoglobin A1c by 26%, 9% and 6%, respectively.^27,28^ Though a meta-analysis of randomized trials showed no effect of vitamin D on risk of T2DM, the authors stated this may be due to suboptimal vitamin D dosing or short time frames of the trials.^29^ Lastly, fish is a source of selenium, which may reduce diabetes risk in individuals with normal plasma selenium concentrations,^30^ but high plasma selenium was recently associated with a 27% increase risk of T2DM^31^ in a Chinese cohort.

Furthermore, some types of fish have considerable amounts of omega-6,^32^ which may affect the association between fish intake and the risk of diabetes. Based on evidence, omega-6 and the ratio of omega-6 to omega-3 fatty acid content of diet play pivotal roles on metabolic status. Omega-3 and omega-6 PUFAs compete for the same enzymes for desaturation and elongation, albeit each has different effects on human health.^32,33^ Accordingly, differences in findings regarding fish intake can be partially explained by this fact.

Seafood often contains persistent organic pollutants (POPs), mercury and other fat-soluble pollutants, which may attenuate the positive effects of fish of fish on human health, or increase the risk of diseases including T2DM.^8^ The effects of pollutants were taken into account in only two of the studies in our meta-analysis.^8,20^ The study by Wallin et al, reported positive correlations (Spearman r: 0.77 for PCB and 0.70 for MeHg) between fish intake and dietary contaminant exposures.^20^ Interestingly though, when adjusting for contaminant intake, the risk of T2DM (HR: 0.79; 95% CI: 0.60-1.04) was not significantly decreased. This is in contrast to a recent cohort study^10^ and a recent meta-analysis^34^ showing positive associations between higher plasma concentrations of PCBs and risk of diabetes. Differences in results among studies may be due to differences in pollutant consumption and exposure. First Nations individuals in Canada had high levels of exposure due to high levels of pollutants in lake fish. Thus, when pollutant exposure is high, the negative impact of exposure may begin at a low fish intake, while beneficial effects of omega-3 likely occur at higher intakes.^10^ Similarly, in our meta-analysis, the small or negligible protective effect of fish intake on T2DM may be due to variation in pollutant exposure, with average PCB concentrations in Asian populations being lower than European populations.^35^ Based on the mean fish/seafood consumed among the included populations in our meta-analysis, protective effects of n-3 fatty acids were likely not sufficient to overcome the negative effects of pollutants.

In the current meta-analysis, shellfish, fish finger, fish roe, and seafood with a mixture of flour, milk and oil were introduced as seafood products other than fish and showed no significant association with risk for diabetes. This may be partially due to lower n-3 PUFA or content in these seafood products, however, subgroup analysis also revealed that fish product intake in Asian populations reduced diabetes risk by 16%, whereas no link was found in European countries. Beyond the above-mentioned increased exposure to environmental contaminants in Europe, other non-modifiable and modifiable factors may also help explain differences among geographic regions. For example, the NOWAC cohort study showed that Norwegian fish eaters were older, had greater BMI, and had a greater proportion of people who were former or current smokers compared to those who did not eat fish, all of which are T2DM risk factors.^8^ Further, Wallin et al reported that seafood products were popularly consumed with high fat sauces like mayonnaise,^10^ adding to saturated fat intake which is another T2DM risk factor.^4^ Also likely higher in European than Asian countries, the regular consumption of a Westernized diet was reported as an important independent risk factor for T2DM.^4^ Although most studies were controlled for several covariates including physical activity, BMI, alcohol intake, age and other food intakes, controlled parameters were not the same across all studies. Additionally, in women (17%) and normal weight (16%) individuals who consumed the highest amount of seafood other than fish, the risk of developing T2DM was lowered. All studies except one,^19^ adjusted findings for BMI, however, only BMI at baseline was reported and BMIs were in the normal weight and overweight ranges. Overweight individuals likely did not see a benefit of fish or seafood intake because a higher BMI can affect insulin function of pancreatic beta-cells, insulin sensitivity and blood glucose concentrations.^36^

Our analysis did not show a significant link between fried fish and risk for T2DM onset. However, only two studies,^21^ reported cooking methods for fish/seafood. The impact of ﬁsh intake on glucose metabolism is likely to differ based on cooking/preparation methods. Deep fried ﬁsh, compared to raw ﬁsh contains lower levels of EPA and DHA^37,38^ and may associate with greater concentrations of contaminants,^39^ as well as an increased production of mutagenic compounds due to high cooking temperature. Combined, these factors likely contribute to insulin resistance.^40^ Patel et al reported that there was an inverse association between non-fried (fresh, frozen, or canned) ﬁsh consumption and T2DM, but not with fried fish. Due to existence of only two studies regarding fried fish, the between-study heterogeneity was moderate and our findings should be used cautiously. Lastly, as reported by Wallin et al, it is possible that fried fish consumption may just be a marker of other unhealthy modifiable behaviors.^20^

In the present meta-analysis, we examined study quality, publication bias and sensitivity analysis that were not considered in the previous similar meta-analyses. However, there were still some limitations. First, limiting the interpretation of the effect of weight or BMI, changes in body weight and BMI throughout the cohort study follow-up periods was not reported in the cohort studies. Second, as cohorts were in normal weight and overweight individuals, the results of this analysis may not apply to obese individuals Also, due to insufficient information about potential environmental contaminants; it was not possible to examine the influence of such toxicants on the association between seafood intake and T2DM. Further, it was not possible to rule out the effects of unmeasured confounding factors. Cooking methods (frying, grilling, stewing) were only reported in 2 cohorts, therefore, cooking methods and side dishes served with fish should be taken into account in future studies examining the link between types of fish and T2DM.^8^ Additionally, as person-year was not reported in the most included studies we could not perform dose-response analysis. Some seafood, including shellfish, is a rich source of cholesterol, which may decrease the insulin secretary capacity of pancreatic beta-cells.^41^ As only one study adjusted results for dietary cholesterol, examining this hypothesis was not possible. Lastly, preserving methods, including salting and drying may impact nutrient content and health effect of fish consumption, however, preserving methods were only reported in one study.^6^

## Conclusion

Our findings indicated that oily fish may have protective effects against the development of T2DM. However, due to the lack of robustness in the findings, results should be interpreted with caution. More cohort studies are needed to draw a certain conclusion. Subgroup analyses suggest that seafood other than fish may reduce the risk of developing T2DM in Asian populations, women and normal weight individuals. Further work is needed to fully understand the negative health effects of the myriad of environmental toxicants.

## Ethical approval

Not applicable.

## Competing interests

The authors declare that they have no competing interests.

## Funding

Tehran University of Medical Sciences (project code: 97-01-161-38071).

## Authors’ contributions

The authors’ responsibilities were as follows: BL, LA designed the research; NN and JH: conducted systematic research; LA, NN: extracted data; NN, NRB, NB: analyzed data; NN, BL and LA: wrote manuscript; All authors: had primary responsibility for the final content of the manuscript; and all authors read and approved the final manuscript. None of the authors reported a conflict of interest related to the study.

## Availability of data and material

All data used in the current study are available from the corresponding author (N.N) on reasonable request.

## Acknowledgments

We would like to express our thankfulness to Tehran University of Medical Sciences for its financial support.

## Supplementary Materials


Supplementary file 1 contains Table S1.
