# Supplementary material for: The association between types of seafood intake and the risk of type 2 diabetes: a systematic review and meta-analysis of prospective cohort studies
Source: Health Promot Perspect. 2019 Aug 6;9(3):164–73. doi: 10.15171/hpp.2019.24 (PMC6717924; doi:10.15171/hpp.2019.24)
Supplement: Supplementary file 1 — contains Table S1. [file hpp-9-164-s001.pdf]

Supplementary file 1

Table S1. The PICO criteria used for the present systematic review

PICO criteria	Description
Patients	Adult subjects
Exposure	“fish” OR “seafood”
Comparison	Subjects in high intake category vs. low intake category of seafood intake
Outcome	Risk of type 2 diabetes mellitus
